# Hypertension in people living with HIV on combined antiretroviral therapy in rural Tanzania

**DOI:** 10.4314/ahs.v23i1.15

**Published:** 2023-03

**Authors:** Silvia Trifirò, Francesco Cavallin, Sabina Mangi, Lawrence Mhaluka, Silvia Maffoni, Stefano Taddei, Giovanni Putoto, Giovanni F Torelli

**Affiliations:** 1 Doctors with Africa CUAMM, Iringa, Tanzania; 2 Azienda Ospedaliero-Universitaria Pisana, Pisa, Italy; 3 Independent statistician, Solagna, Italy; 4 Tosamaganga Council Designated Hospital, Iringa, Tanzania; 5 University of Pavia, Italy; 6 University of Pisa, Italy; 7 Doctors with Africa CUAMM, Padua, Italy; 8 Doctors with Africa CUAMM, Dar Es Salaam, Tanzania; 9 Policlinico Umberto I, Rome, Italy

**Keywords:** HIV, Hypertension, sub-Saharan Africa

## Abstract

Exposure to anti-retroviral therapy in HIV infection has been associated with hypertension, but whether and to what extent HIV-related factors and anti-retroviral treatment contribute to hypertension is not well defined; in addition, data are particularly scarce in Sub-Saharan Africa.

Aim of the study was to investigate prevalence and awareness of hypertension in a cohort of people living with HIV (PLWHIV) on anti-retroviral therapy in rural Tanzania, and to identify possible predictors of hypertension.

A cross-sectional study on hypertension in PLWHIV was conducted at Tosamaganga District Hospital, Iringa Region, Tanzania. Subjects on anti-retroviral therapy, age 26-80 years and with monthly attendance to the HIV clinic, were considered eligible.

A total number of 242 patients were included in the analysis. Sixty-two subjects (26%) had hypertension, the majority (77%) of them not aware of the condition and/or not on treatment. Older age, higher BMI and lower baseline T-CD4 count were predictors of hypertension at multivariate analysis. The results of the study suggest that hypertension screening should become part of ordinary care of PLWHIV in Tanzania, particularly in subjects with more severe immunosuppression. Leveraging already existing HIV services could be an option to prevent the burden of non-AIDS complication and related deaths.

## Introduction

HIV infection is one of the major global public health concerns, with around 38 million people estimated to be HIV positive in 2018, of whom around two thirds living and aging in Sub-Saharan Africa (SSA) 1. The increasing use of combined anti-retroviral therapy (cART) has shifted the course of the infection to a chronic condition, substantially increasing the survival of people living with HIV (PLWHIV) and posing the new challenge of non-AIDS-related chronic diseases, such as cardiovascular diseases (CVDs) and other chronic non communicable diseases (NCDs) 2–4. These conditions generally occur due to aging and to the nutrition transition that SSA countries are facing, together with demographic, urban and economic development, leading to a shift of the nutritional status from predominant undernourishment to higher rates of overweight and obesity 5.

Overall, these factors represent additional and long-term burdens for fragile health care services in low-resource settings, historically oriented toward reproductive health and acute communicable diseases 6.

PLWHIV have been demonstrated to be at high risk of CVDs 7–9, and hypertension represents an important cardiovascular risk factor in these patients 1. HIV and cART are associated with CVDs in several pathophysiological pathways. HIV infection per se has been demonstrated to accelerate inflammatory processes known to promote atherosclerosis and hypertension, such as endothelial dysfunction and thrombosis 11. Exposure to cART has been associated with hypertension 12 and patients receiving cART, especially those based on protease-inhibitors (PI), seem more prone to develop overweight, obesity and metabolic derangements, providing a metabolic profile at increased risk of hypertension 13. In addition, well-known risk factors for hypertension, such as smoking, alcohol intake and physical inactivity, are frequently reported in PLWHIV 14. On the whole, PLWHIV on cART seem a category at higher risk for hypertension, when compared both to the general population and to cART naïve subjects^14^. However, epidemiological data regarding hypertension among PLWHIV in SSA are scarce 15. Furthermore, whether and to what extent HIV-related factors and cART contribute to hypertension is not well-defined, with contradictory and often inconsistent data. Besides, vertical international programs on HIV and AIDS do not always focus on NCDs in low-resources setting.

The aim of this study was to investigate prevalence and awareness of hypertension in a cohort of PLWHIV on cART in rural Tanzania, and to identify possible predictors of hypertension among clinically relevant factors.

## Materials and methods

### Study design

This was a retrospective cross-sectional study on hypertension in PLWHIV attending the local HIV clinic at Tosamaganga Hospital, Tanzania from November 2017 to February 2018. The study was conducted according to Helsinki Declaration principles, and approved by the Hospital Management Team, who waived the need for patient written consents, given the retrospective nature of the study and the use of anonymized data.

### Setting

The study was conducted at the HIV clinic of Tosamaganga Hospital in the Iringa District Centre, Tanzania. Tosamaganga Hospital is a district designated hospital located in a rural area in South-western Tanzania and serves approximately 260,000 people. Tosamaganga Hospital has a capacity of 164 beds and an outpatient area that includes general ambulatory services and the HIV clinic. HIV prevalence rates varies largely across the country and, while a relatively low HIV prevalence is observed in some areas of Northern Tanzania, particularly high rates of infection are reported in the South, up to 11.3% in Iringa region.

### Institutional background

Doctors with Africa CUAMM (Collegio Universitario Aspiranti Medici Missionari) is an Italian Non-Governmental Organization (NGO) which has been supporting health service delivery in Africa for 70 years 16. CUAMM started working in Tanzania in 1968, in support of not-for-profit facilities in Njombe region, and has now widened its activities to 6 regions, currently working in 105 health centers and two hospitals across the country. CUAMM has been supporting Tosamaganga Hospital since 1988.

### Patients

PLWHIV attending the HIV clinic for clinical follow up and for monthly dispensation of cART from November 2017 to February 2018 were retrospectively evaluated for inclusion in the study. PLWHIV, aged between 26 and 80 years were eligible for inclusion. Exclusion criteria were pregnancy, lactation or unavailability of variables of interest.

### Variables

Demographical data, social information and past medical history were retrieved from hospital records. Biometric data, blood pressure and capillary blood glucose were also collected from recorded data. Hypertension was defined as a clinical diagnosis with blood pressure (BP) ≥ 140/90 mmHg, as defined by WHO 17. Blood pressure was taken using a manual sphygmomanometer. Height was taken with a portable stadiometer, in centimetres. Weight was taken with an analogic weighting scale. Body mass index (BMI) was calculated as weight (kg) divided by the square of height (m^2^) and categorized using the standardized definition of the WHO: <18.5 kg/m^2^ as underweight, 18.5–24.9 kg/m^2^ as normal weight, 25.0–29.9 kg/m^2^ as overweight; and >30 kg/m^2^ as obesity 18. Waist circumference measurements were done with a tape at the approximate midpoint between the lower margin of the last palpable rib and the top of the iliac crest as per WHO guidelines. Central obesity was defined as waist circumference > 88 cm for females, >102 cm for males 19. Diabetes was diagnosed with fasting glucose ≥ 126 mg/dl and/or random blood glucose ≥ 200 mg/dl, as per WHO definitions 20.

### Statistical analysis

Continuous variables were summarized as median and interquartile range, and categorical variables as number and percentage. Association between categorical variables was evaluated using Fisher's test or Chi Square test. Association between binary variables and continuous variables was evaluated using Mann-Whitney test. A logistic regression model was estimated to identify the independent predictors of hypertension among clinically relevant factors. Some variables of interest were not included in the model due to small occurrence (diabetes, cardiac disease and family history for hypertension), while duration of HIV infection and duration of cART were not included due to high collinearity with age.

Initial model included age, BMI, waist circumference, WHO stage and T-CD4 cells count at diagnosis. Model selection was performed by minimizing the Akaike information criterion (AIC): waist circumference and WHO stage at diagnosis were removed from the model due to inflation of AIC. Model performance was evaluated with internal validation (c-index) and calibration (calibration-in-the-large and calibration slope) using bootstrap methods and showed moderate validation (c-index 0.738) and good calibration (calibration-in-the-large -0.018 and calibration slope 1.015).

All tests were 2-sided and a p-value less than 0.05 was considered statistically significant. Data analysis was performed using and R 3.5 (R Foundation for Statistical Computing, Vienna, Austria) 21.

## Results

A total number of 360 patients attended the HIV clinic November 2017 to February 2018. One hundred eighteen patients were excluded from the analysis (six did not meet age criteria and 112 had no information on blood pressure).

Finally, 242 patients (median age 43 years, IQR 38-50; 98 males and 146 females) were included in the analysis. Patients' characteristics are shown in Table 1.

**Table 1:** Patient characteristics

Variables	All patients	No hypertension	Hypertension	p-value
N of subjects	242	180	62	-
Age, years ^a^	43 (38–50)	42 (37–50)	49 (42–57)	<0.0001
Male: Female (%)	96:146 (40:60)	70:110 (39:61)	26:36 (42:58)	0.78
Education level: None Primary Secondary or above	25 (10.3) 198 (81.8) 19 (7.9)	16 (8.9) 151 (83.9) 13 (7.2)	9 (14.5) 47 (75.8) 6 (9.7)	0.34
Farmers	222 (91.7)	164 (91.1)	58 (93.5)	0.74
Personal insurance holders	17 (7.0)	9 (5.0)	8 (12.9)	0.07
Tobacco smoking: No Former Current	204 (84.2) 14 (5.8) 24 (10.0)	153 (85.0) 9 (5.0) 18 (10.0)	51 (82.2) 5 (8.1) 6 (9.7)	0.67
Alcohol intake: No Former Current	144 (59.5) 55 (22.7) 43 (17.8)	109 (60.6) 38 (21.1) 33 (18.3)	35 (56.5) 17 (27.4) 10 (16.1)	0.59
History of stroke	3 (1.2)	1 (0.6)	2 (3.2)	0.10
History of cardiac disease	8 (3.3)	1 (0.6)	7 (11.3)	0.0002
Fasting BG, mg/dl ^a^	92 (83–99)	91 (82–98)	93 (86–106)	0.07
Random BG, mg/dl ^a^	91 (85–99)	90 (84–98)	95 (88–99)	0.14
Diabetes ^b^	10 (4.2)	3 (1.7)	7 (11.3)	0.004
Family history of hypertension	17 (7.0)	7 (3.9)	10 (16.1)	0.003
BMI, kg/m^2 ac^	21.6 (19.8–24.3)	21.3 (19.6–23.8)	23.3 (20.5–27.0)	0.002
BMI categories, kg/m^2: c^ < 18.5 18.5–24.9 25.0–29.9 >30	26 (11.0) 166 (70.0) 35 (14.8) 10 (4.2)	19 (10.7) 135 (76.3) 20 (11.3) 3 (1.7)	7 (11.7) 31 (51.7) 15 (25.0) 7 (11.7)	0.0002
Waist circumference, cm ^ad^	78 (73–85)	78 (73–83)	83 (75–94)	0.003
Central obesity	19 (7.9)	9 (5.0)	10 (16.1)	0.01

Legend: Data expressed as No. (%) or ^a^ median (IQR). Fasting and random blood glucose (BG) were available in 158 and 86 patients, respectively. Data not available in ^b^3, ^c^5, ^d^8 patients.

Sixty-two patients (25.6%) had hypertension and 48 of them (77%) were not aware of the condition and/or were not on treatment. Ten subjects (4.2%) had diabetes. Thirty-five patients (14.8%) were overweight, 10 (4.2%) were obese and 19 patients (7.9%) had central obesity.

Hypertension was associated with older age (p<0.0001), previous history of cardiac disease (p=0.0002), diabetes (p=0.004), family history for hypertension (p=0.003), higher BMI (p=0.002) and larger waist circumference (p=0.003) (Table 1).

Median known duration of HIV infection was 6 years (IQR 3-9 years) and median duration of cART was 5 years (IQR 2-8). Information on HIV infection and cART history is reported in Table 2. Overall, cART was started at median 76 days (IQR 20-278) after HIV diagnosis.

**Table 2:** HIV infection and cART history

Variables	All patients	No hypertension	Hypertension	p-value
N of patients	242	180	62	-
Known duration of HIV infection, years ^ab^	6 (3–9)	6 (3–9)	8 (3–10)	0.04
WHO clinical stage at diagnosis: ^c^ I II III IV	44 (21.1) 41 (19.7) 92 (44.3) 31 (14.9)	40 (25.8) 30 (19.4) 63 (40.6) 22 (14.2)	4 (7.5) 11 (20.8) 29 (54.7) 9 (17.0)	0.04
T-CD4, cells/µl ^a^ At diagnosis ^d^ At last visit ^e^	213 (113–314) 518 (382–730)	219 (123–329) 509 (382–745)	153 (65–279) 546 (378–663)	0.01 0.53
cART start after diagnosis, days ^af^	76 (20–278)	79 (21–294)	54 (15–212)	0.43
Duration of cART, years ^ag^	5 (2–8)	4 (2–8)	7 (3–9)	0.009
Number of cART lines, ^h^ 1 line 2 lines 3 lines 4 lines	187 (79.2) 34 (14.4) 12 (5.1) 3 (1.3)	142 (81.1) 23 (13.1) 8 (4.6) 2 (1.1)	45 (73.8) 11 (18.0) 4 (6.6) 1 (1.6)	0.58
cART ^h^ Not PI-based PI-based	217 (91.9) 19 (0.1)	161 (92.0) 14 (8.0)	56 (91.8) 5 (8.2)	0.99

Legend: Data expressed as No. (%) or ^a^ median (IQR). Data not available in ^b^7, ^c^34, ^d^28, ^e^25, ^f^11, ^g^9, ^h^6 patients.

All the patients were on cART. The majority of patients were receiving a first line regimen (79.2%) and the most common cART combination was not PI-based (91.9%) vs PI-based (17.1%). The single tablet, co-formulated combination of tenofovir disoproxil fumarate (TDF), lamivudine (3TC) and efavirenz (EFV) was the most common regimen (60%), followed by the single tablet containing TDF, emtricitabine (FTC) and EFV (14%). Lopinavir/ritonavir (LPV/r) was the most prescribed PI (6.2%). Median T-CD4 cells count increased from 213 cells/µl (IQR 113-314) at diagnosis (baseline T-CD4 cells count) to 518 cells/µl (IQR 382-730) at the last visit (p<0.0001). Hypertension was associated with more advanced WHO clinical stage at diagnosis (p=0.04), longer duration of HIV infection (p=0.04) and longer exposure to cART (p=0.009) (Table 2). Hypertension was also associated with lower baseline T-CD4 cells count (p=0.01) but neither with T-CD4 cells count at last visit (p=0.53) nor with PI use (Table 2).

Multivariable analysis of hypertension was performed using a logistic regression model. Older age (OR 1.06, 95% CI 1.02 to 1.10) and higher BMI (OR 1.15, 95% CI 1.06 to 1.25) were associated with increased odds of hypertension, while higher T-CD4 cells count at diagnosis (OR 0.73, 95% CI 0.56 to 0.92) was associated with decreased odds of hypertension (Table 3).

**Table 3:** Multivariable analysis of hypertension

Variables	p-value	OR (95% CI)
Age, years	0.001	1.06 (1.02 to 1.10)
BMI, kg/m^2^	0.002	1.15 (1.06 to 1.25)
T-CD4 at diagnosis, x100 cells/µl	0.01	0.73 (0.56 to 0.92)

## Discussion

Our findings showed a considerable prevalence of hypertension, with high rate of unawareness, in a cohort of PLWHIV on cART in rural Tanzania. Hypertension prevalence in our study was high and comparable to available data in SSA ranging from 16% to 30% according to the study region 22–26. The rate of hypertension unawareness was similar to data in literature as well, underlying the high burden of asymptomatic and untreated hypertension in PLWHIV in Tanzania.

Older age, higher BMI and lower baseline T-CD4 cells count were associated with increased likelihood of hypertension at multivariable analysis. Longer duration of HIV infection and longer exposure to cART might also be associated with hypertension, but multicollinearity of these parameters with age prevented any definitive conclusions. Well-known traditional cardiovascular risk factors such as older age and higher BMI were identified as independent risk factors for hypertension, same as observed in HIV-negative people.

Furthermore, lower baseline T-CD4 cells count, were found associated with hypertension, in agreement with findings of some previous studies [27-30]. On the whole, low-grade chronic inflammation, deriving both from overweight with excess in visceral adipose tissue and long-standing HIV infection, could take part in the inflammatory processes that lead to hypertension, as reported in literature 31. The specific mechanism of interaction between HIV infection and hypertension seems chronic immune activation, which is recognized to be pro-inflammatory and pro-atherosclerotic and the basis of T-CD4 depletion. Thus, T-CD4 depletion could represent the epiphenomenon of chronic processes that lead to both to immunosuppression and hypertension 32.

This hypothesis is contradictory with some other studies reported in literature 33–34 but similar findings from large multinational cohorts of PLWHIV on cART corroborate the hypothesis that low T-CD4 cells count are a HIV-related predictor of hypertension 35.

Our findings support an inverse relationship between baseline T-CD4 cells count and hypertension 27–30, and suggest a possible association between blood pressure and duration of HIV infection and length of cART exposure. Older patients and/or patients with overweight, long standing HIV infection or low T-CD4 levels should be particularly targeted for frequent blood pressure monitoring and the identification of other cardiovascular risk factors to encourage lifestyle modification and early treatment.

The study has some limitations. First, it is a single-center study in rural Sub-Saharan Africa, thus the generalizability of the findings is limited to similar settings. Second, the retrospective data collection limits the possibility of analysing follow-up data. Furthermore, the majority of subjects were exposed to a single cART (tenofovir, efavirenz and lamivudine), which is not frequently associated to cardio-metabolic risk, and none of them was cART naïve, thus limiting any comparisons of effects of cART per se or alternative cART not in use in Tosamaganga on blood pressure. Finally, the scarce availability of diagnostic resources at Tosamaganga Hospital could have hampered the possibility to diagnose other NCDs, such as cardiovascular diseases, diabetes, kidney diseases and dyslipidemias, and its relationship with HIV infection and immunosuppression.

Our findings could aid governmental and international health care actors in the assessment of services requiring support and implementation at regional level in Tanzania, such as active screening for hypertension and dedicated treatment programs among PLWHIV living in Iringa region. This is even more important when considering the significantly higher prevalence of HIV infection in Iringa region as compared to national data.

Aging and exposure to cART are likely to configure among PLWHIV chronic health care needs than overlap to the ones of general population, and leveraging HIV clinics for NCDs could be an option to face the double burden of NCDs and HIV in Tanzania 36. Ordinary HIV follow-up could be an opportunity for screening, counseling and managing hypertension and the other non-infectious comorbidities found in our cohort of PLWHIV on cART, such as overweight. An adequate management of weight and eating habits could therefore prevent or contribute at reducing the increase in BP in these subjects, thus reducing the cardio-metabolic burden and related complications.

On the whole, we believe in a comprehensive approach to the chronic care of HIV subjects on cART. Vertical projects aiming exclusively at HIV care may miss critical aspects to improve overall health of PLWHIV on cART in Tanzania, such as hypertension and excessive body weight.
